# Supplementary material for: Structural engineering of chimeric antigen receptors targeting HLA-restricted neoantigens
Source: Nat Commun. 2021 Sep 6;12:5271. doi: 10.1038/s41467-021-25605-4 (PMC8421441; doi:10.1038/s41467-021-25605-4)
Supplement: Supplementary file 1 — Supplementary Information [file 41467_2021_25605_MOESM1_ESM.pdf]

Structural engineering of chimeric antigen receptors targeting HLA-restricted neoantigens

Michael S. Hwang[1,2,3,14]†, Michelle S. Miller[2,4,5,15]†, Puchong Thirawatananond[4], Jacqueline Douglass[1,2,3], Katharine M. Wright[2,4,5], Emily Han-Chung Hsiue[1,2,3], Brian J. Mog[1,2,3,6], Tihitina Y. Aytenfisu[4], Michael B. Murphy[7], P. Aitana Azurmendi[4], Andrew D. Skora[1,2,16], Alexander H. Pearlman[1,2,3], Suman Paul[1,2,3,8], Sarah R. DiNapoli[1,2,3], Maximilian F. Konig[1,2,3,9], Chetan Bettegowda[1,3,8,10], Drew M. Pardoll[5,8], Nickolas Papadopoulos[1,3,8,11,12], Kenneth W. Kinzler[1,3,5,8,12], Bert Vogelstein[1,2,3,5,8,11,12]*, Shibin Zhou[1,3,5,8]*, Sandra B. Gabelli[4,8,13]*

† These authors contributed equally to this work
* Corresponding authors

Affiliations:

[1] Ludwig Center, Sidney Kimmel Comprehensive Cancer Center, Johns Hopkins University School of Medicine, Baltimore, MD, USA

[2] Howard Hughes Medical Institute, Chevy Chase, MD, USA

[3] Lustgarten Laboratory for Pancreatic Cancer Research, Sidney Kimmel Comprehensive Cancer Center, Johns Hopkins University School of Medicine, Baltimore, MD, USA

[4] Department of Biophysics and Biophysical Chemistry, Johns Hopkins University School of Medicine, Baltimore, MD, USA

[5] Bloomberg~Kimmel Institute for Cancer Immunotherapy, Sidney Kimmel Comprehensive Cancer Center, Baltimore, MD, USA

[6] Department of Biomedical Engineering, Johns Hopkins University, Baltimore, MD, USA

[7] Cytiva, Marlborough, MA, USA

[8] Department of Oncology, Johns Hopkins University School of Medicine, Baltimore, MD, USA

[9] Division of Rheumatology, Department of Medicine, Johns Hopkins University School of Medicine, Baltimore, MD, USA

[10] Department of Neurosurgery, Johns Hopkins University School of Medicine, Baltimore, MD, USA

[11] Department of Pathology, Johns Hopkins University School of Medicine, Baltimore, MD, USA

[12] Sol Goldman Pancreatic Cancer Research Center, Johns Hopkins University School of Medicine, Baltimore, MD, USA

[13] Department of Medicine, Johns Hopkins University School of Medicine, Baltimore, MD, USA

[14] Present address: Genentech, Inc., South San Francisco, CA, USA
[15] Present address: Walter and Eliza Hall Institute of Medical Research, Parkville, VIC, AUS
[16] Present address: Lilly Biotechnology Center, Eli Lilly and Co, San Diego, CA, USA
To whom correspondence should be addressed:
Email: gabelli@jhmi.edu (S.B.G.), sbzhou@jhmi.edu (S.Z.); vogelbe@jhmi.edu (B.V.)

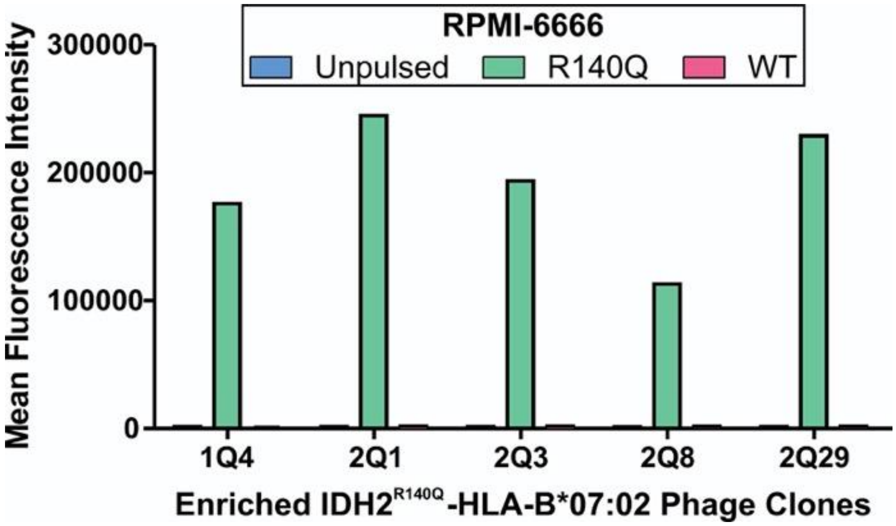

Supplementary Fig. 1. Characterization of IDH2^{R140Q}-HLA-B*07:02 scFvs identified by phage display technology. To evaluate their specificity, candidate IDH2^{R140Q}-HLA-B*07:02 phage clones were used to stain unpulsed RPMI-6666 cells, or RPMI-6666 cells pulsed with IDH2^{R140Q} or IDH2^{WT} peptides, followed by flow cytometry. Source data are provided as a Source Data file.

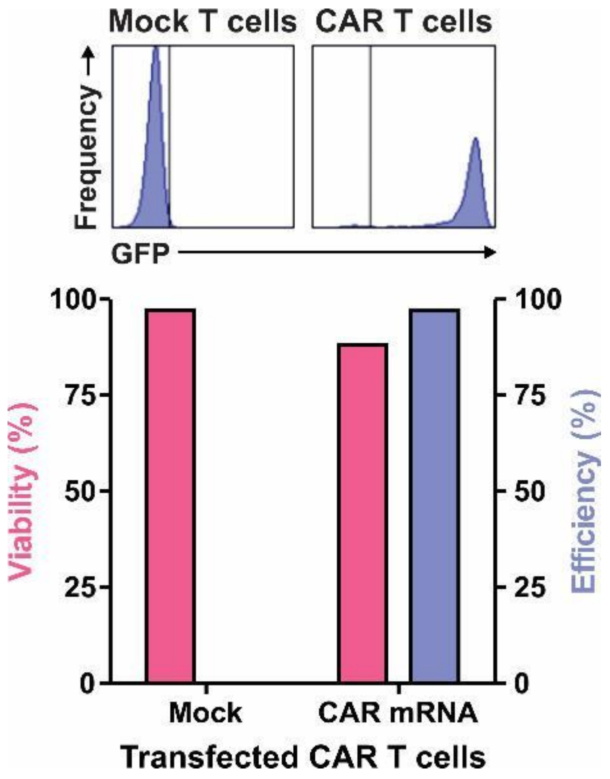

Supplementary Fig. 2. Optimization of mRNA electroporation into primary human T cells. Efficiency of CAR mRNA transfection of primary human T cells (top and bottom panels) and post-electroporation viability of transfected T cells (bottom panel), as evaluated by flow cytometry. "Mock" indicates T cells electroporated with no mRNA. Source data are provided as a Source Data file.

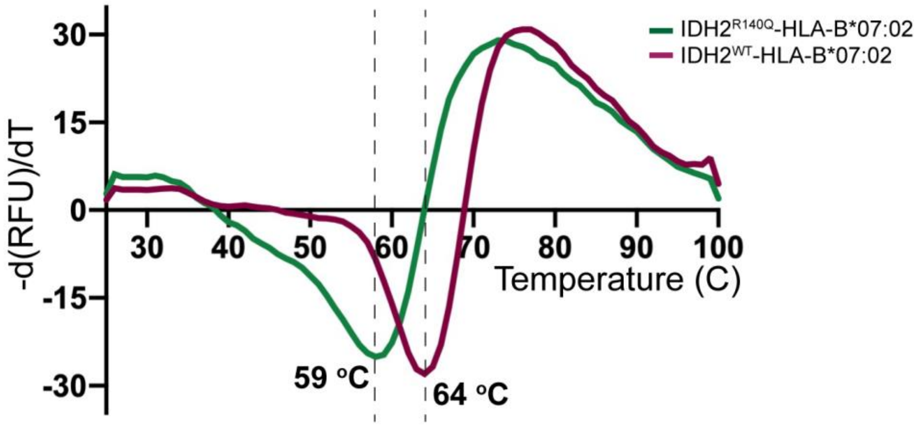

Supplementary Fig. 3. Thermal stability of IDH2^{WT}- and IDH2^{R140Q}-HLA-B*07:02. Differential scanning fluorimetry analysis of IDH2^{WT}-HLA-B*07:02 (raspberry) and IDH2^{R140Q}-HLA-B*07:02 (dark green) showing the negative derivative, -d(RFU)/dT, of relative fluorescence unit (“RFU”) vs. temperature. The melting temperatures correspond to the negative peak maximums of the first derivative (dashed lines) and are indicated in the corresponding graphs by the noted temperature. Source data are provided as a Source Data file.

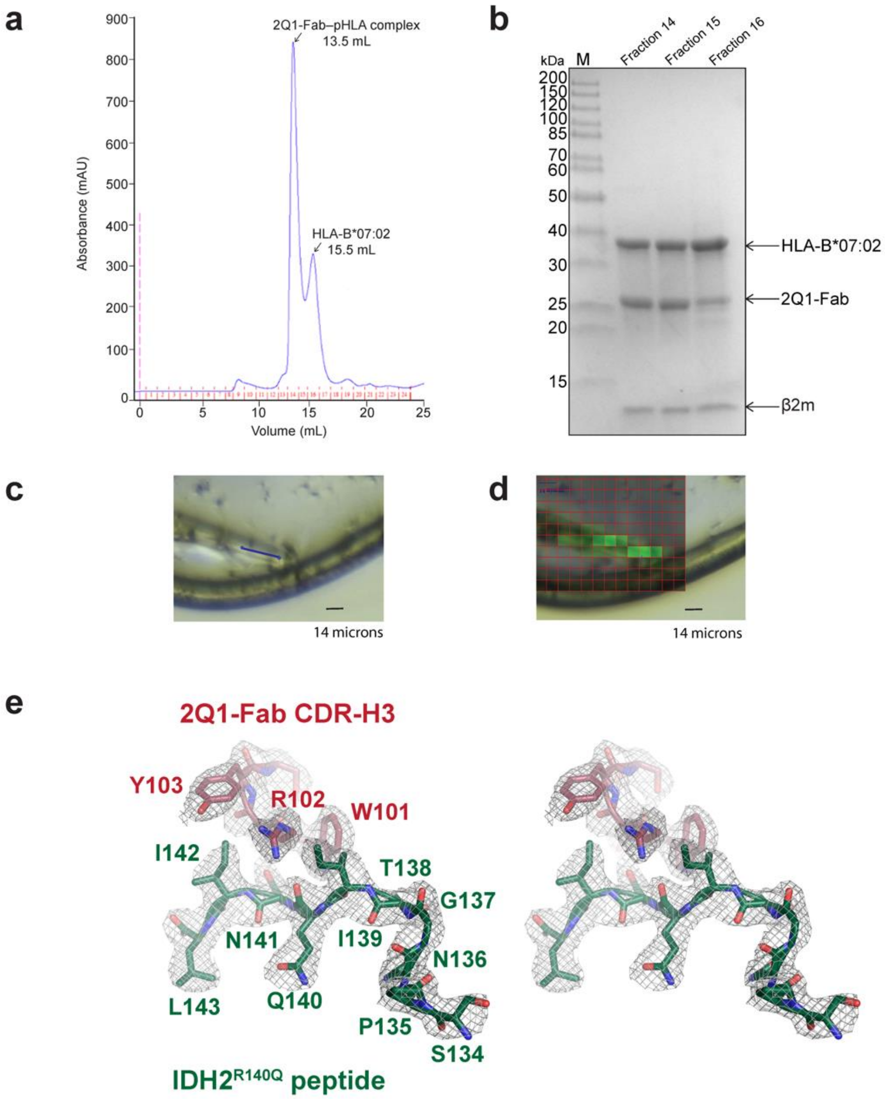

Supplementary Fig. 4. Purification of IDH2^{R140Q}-HLA-B*07:02 in complex with the 2Q1-Fab. a Size-exclusion chromatogram of the pHLA-B*07:02 in complex with the 2Q1-Fab. Protein was monitored by A280 nm showing a major peak (~100 kDa), N=2 **b** Coomassie-stained gradient SDS-PAGE gel of the eluted fractions 14-16 (13-17 mL) from (**a**) denoting 2Q1-Fab, β2M and HLA-B*07:02. In the gel, fractions 14 and 15 show similar intensities of IDH2^{R140Q}-HLA-B*07:02 and 2Q1-Fab, signifying complex, whereas fraction 16 (seen as a separate peak, A) has a significantly lower concentration of 2Q1-Fab, indicating the second peak is predominantly IDH2^{R140Q}-HLA-B*07:02, N=3 **c** Collected microcrystal of the 2Q1-Fab/IDH2^{R140Q}-HLA-B*07:02 complex with a width of ~14 microns and the vector used for data collection marked as a blue line (35 microns), N=6. **d** as (**c**) with the heat map generated by raster-scanning the microcrystal, N=6. **e** 2Fo-Fc cross-eye stereo electron density map of IDH2^{R140Q} peptide and a region of the CDR-H3 of the 2Q1-Fab (σ=1). Source data are provided as a Source Data file.

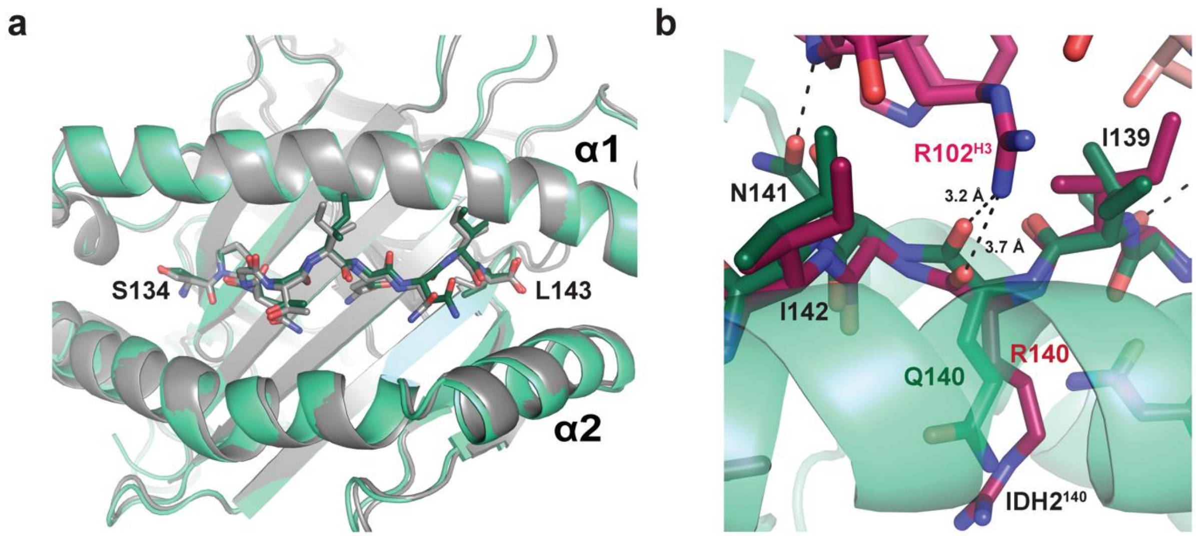

Supplementary Fig. 5. The peptide-binding helices α1 and α2 do not move upon 2Q1-Fab binding. a The 2Q1-Fab-bound pHLA is shown in green, while the unbound structure is shown in gray. There is no significant movement of the helices upon binding of the 2Q1-Fab. **b** Zoomed in view of the structural alignment the IDH2^{WT}-HLA-B*07:02 (PDB ID 6UJ8, raspberry) and 2Q1-Fab/IDH2^{R140Q}-HLA-B*07:02 (PDB ID 6UJ9, green) structures highlighting the essential interaction between 2Q1-Fab R102 residue and the backbone carbonyl of the epitope of interest IDH2^{140}. Measured distances are depicted as dashed lines and labeled.

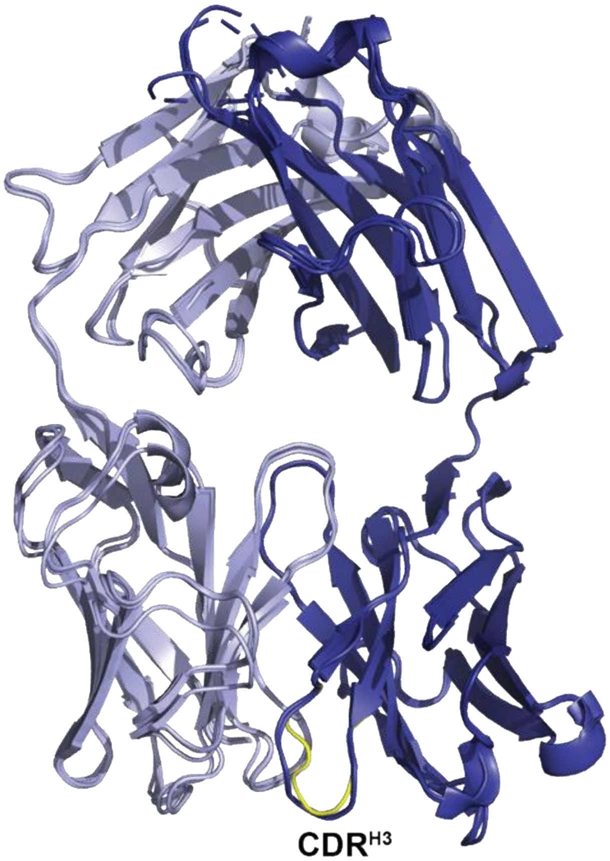

Supplementary Fig. 6. Structure of the free 2Q1-Fab. Structural alignment of the four 2Q1-Fab copies in the asymmetric unit. The light chain is shown in light blue, and the heavy chain in deep blue. The conformation of CDR-H3 was different in one of the four copies, highlighted in yellow.

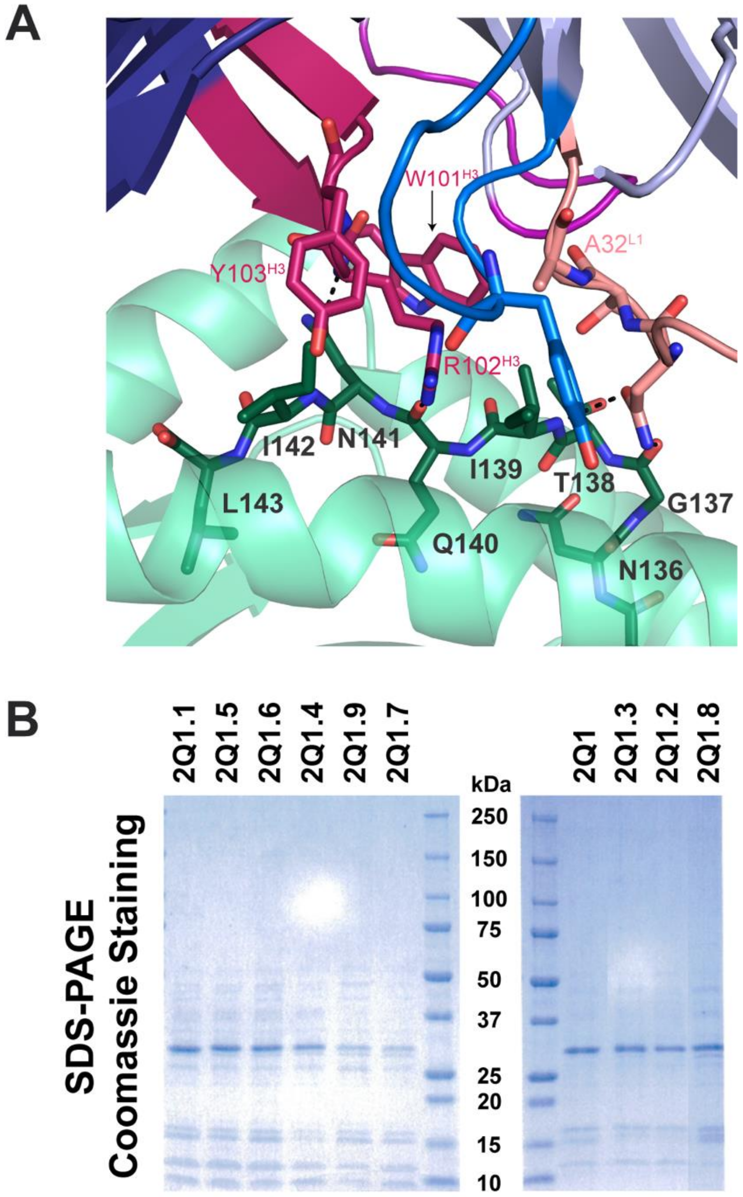

Supplementary Fig. 7. Recombinant scFv expression of 2Q1 variants. a Zoomed view about 180° from Fig. 3A (insert) showing the residues of the 2Q1-Fab involved in the recognition of IDH2^{R140Q}-HLA-B*07:02. The residues mutated in the 2Q1 variants (A32^{L1} W101^{H3}, R102^{H3} and Y103^{H3}) are labeled. **b** Site-directed mutagenesis on the parental 2Q1 scFv was performed with the indicated amino acid substitutions, recombinantly expressed in *E. coli*, purified using nickel chromatography, resolved on an SDS-PAGE gel, and stained with Coomassie dye. Horizontal numbers depicted indicate molecular weight in kDa. Data are representative of three independent experiments. Source data are provided as a Source Data file.

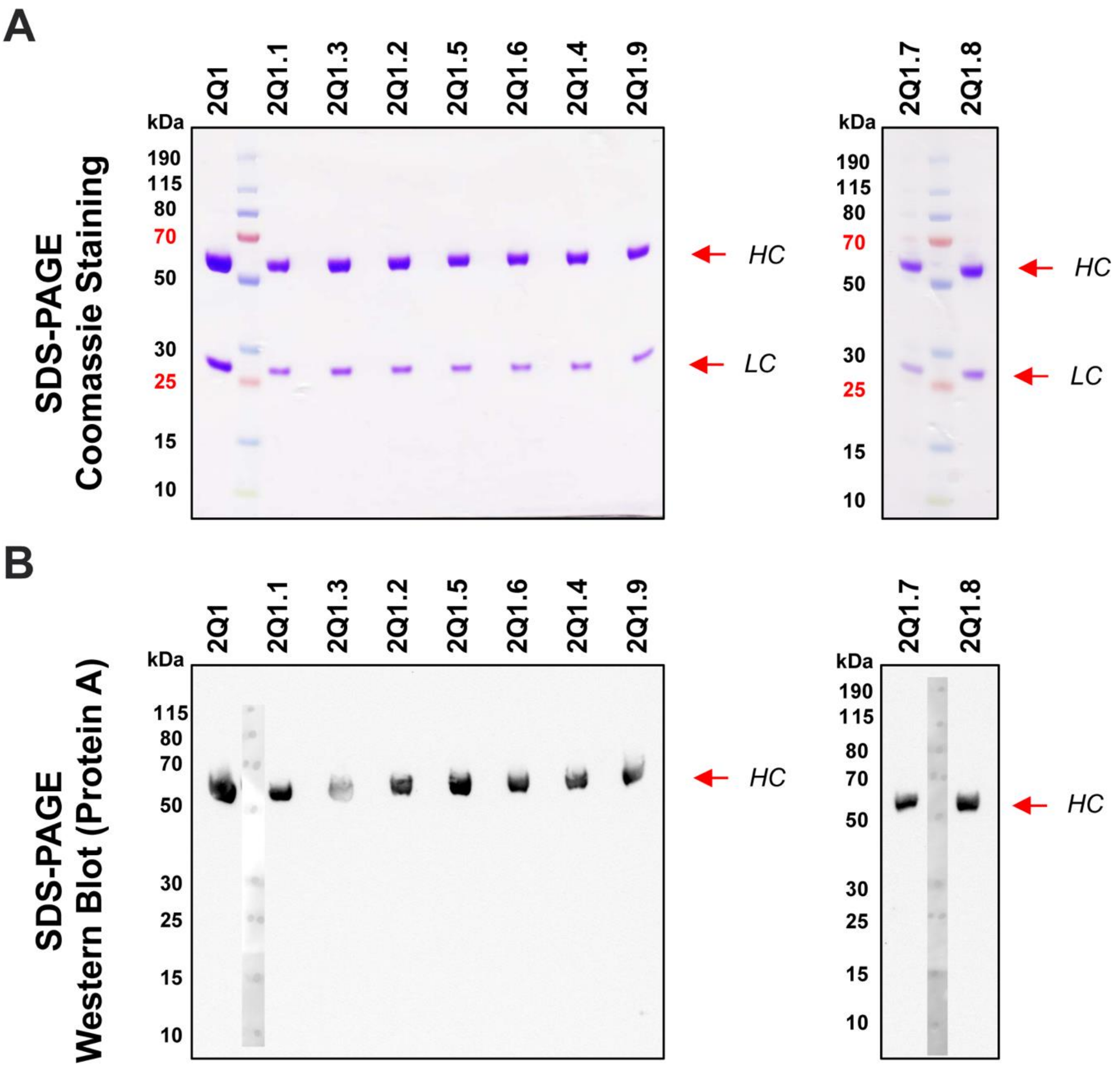

Supplementary Fig. 8. Mammalian IgG1 expression of 2Q1 variants. Site-directed mutagenesis on the parental 2Q1 clone was performed with the indicated amino acid substitutions, expressed in mammalian Expi293 cells, purified with Protein A, resolved on an SDS-PAGE gel, and **a** stained with Coomassie dye. Data are representative of two independent experiments; or **b** immunoblotted with Protein A. HC, heavy chain; LC, light chain. Horizontal numbers depicted indicate molecular weight in kDa. Data are representative of two independent experiments. Source data are provided as a Source Data file.

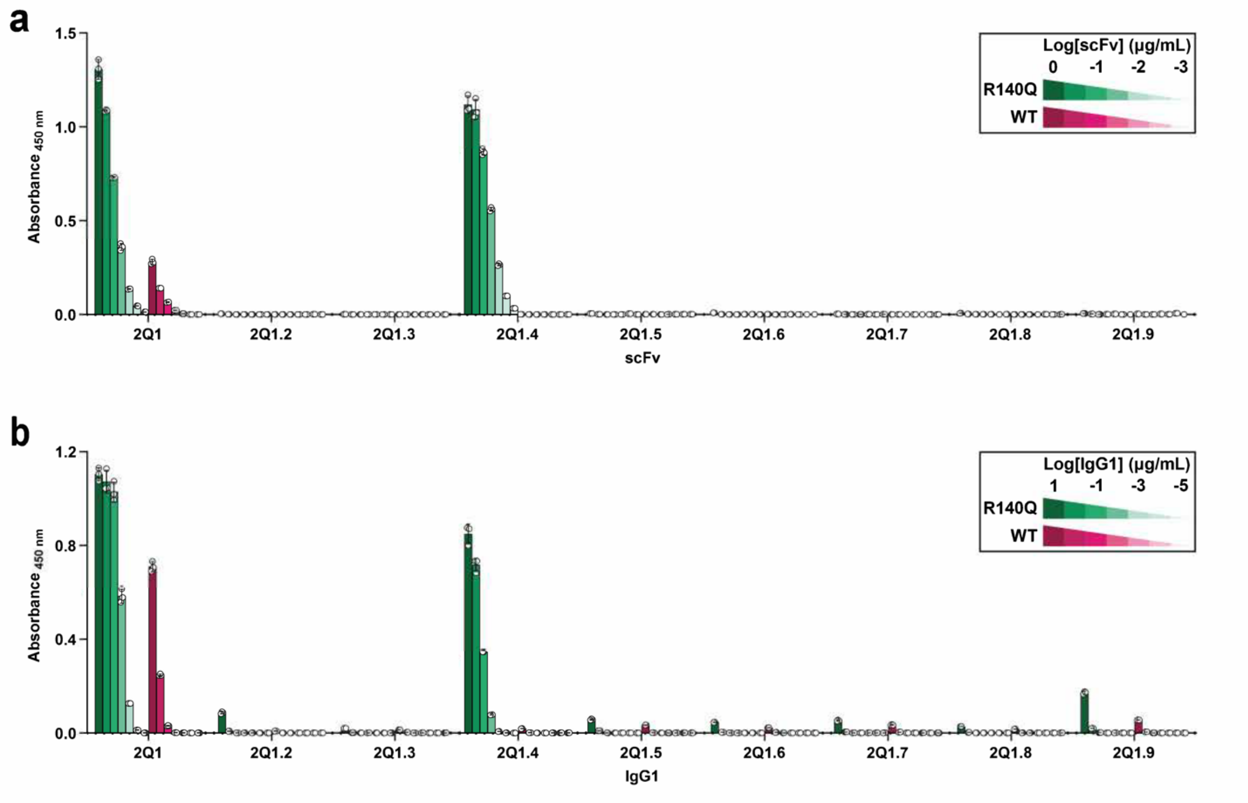

Supplementary Fig. 9. Data distribution of Fig. 5. a Binding of 2Q1 and its variant scFvs to IDH2^{R140Q} or IDH2^{WT} pHLA monomers was assessed by ELISA. Bars represent the mean of three technical replicates ± SD, while open circles represent the individual data points. **b** Binding of 2Q1 and its variant IgG1s to IDH2^{R140Q} or IDH2^{WT} pHLA monomers was assessed by ELISA. Bars represent the mean of three technical replicates ± SD, while open circles represent the individual data points.

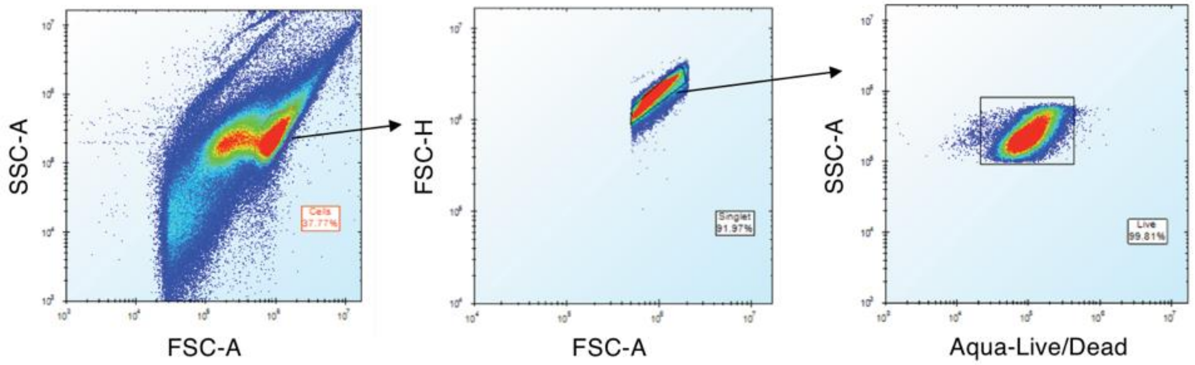

Supplementary Fig. 10. Gating strategy used for Fig. 5c, d. Viable singlet cells were selected for analysis.

Supplementary Table 1. Summary of tested clones. A list of original IDH2^{R140Q}-HLA-B*07:02-targeting clones and subclone variants of 2Q1 with single amino acid substitutions are shown. TRIM, trinucleotide mutagenesis; H, heavy chain; L, light chain.

Clone Name	Originating Phage Library	Amino Acid Substitution (IgG1 Chain)	Parental Clone
1Q4	1st Generation (NNK)	—	—
2Q1	2nd Generation (TRIM)	—	—
2Q3	2nd Generation (TRIM)	—	—
2Q8	2nd Generation (TRIM)	—	—
2Q29	2nd Generation (TRIM)	—	—
2Q1.1	—	R102A (H)	2Q1
2Q1.2	—	W101H (H)	2Q1
2Q1.3	—	W101R (H)	2Q1
2Q1.4	—	Y103H (H)	2Q1
2Q1.5	—	Y103K (H)	2Q1
2Q1.6	—	Y103R (H)	2Q1
2Q1.7	—	A32F (L)	2Q1
2Q1.8	—	A32H (L)	2Q1
2Q1.9	—	A32W (L)	2Q1

Supplementary Table 2. Kinetics and affinity of 2Q1- and 2Q1.4-Fab binding to mutant and WT pHLA-B7 as determined by SPR. nd, not determined; NB, no binding.

	IDH2^{R140Q}-HLA-B*07:02			IDH2^{WT}-HLA-B*07:02		
	k_{on} ($M^{-1}s^{-1}$)	k_{off} (s^{-1})	K_D (nM)	k_{on} ($M^{-1}s^{-1}$)	k_{off} (s^{-1})	K_D (nM)
2Q1-Fab	1.59×10^6	9.66×10^{-2}	44	1.31×10^3	1.11×10^{-3}	846
2Q1.4-Fab	4.91×10^3	8.60×10^{-4}	175	nd	nd	NB

Supplementary Table 3. Contacts between the 2Q1-Fab and IDH2^{R140Q}-HLA-B*07:02.
Total bonds were calculated using a 4 Å cutoff which includes both hydrogen bonds and van der Waals interactions as calculated using CONTACT in the CCP4 suite[95]. BSA, buried surface area; HC, V_H domain; LC, V_L domain; pep, HLA presented peptide.

	2Q1-Fab/IDH2^{R140Q}-HLA-B*07:02 (PDB ID 6UJ9)
Affinity (K_D)	44 nM
Total bonds	159
Peptide bonds **(bold ≥10)**	4, 5, **6**, 7, **8**, 9
Peptide bonds	43 (27%)
Bonds from HC	28
Bonds from LC	15
HLA bonds	116
Bonds from HC	78
Bonds from LC	38
BSA	
BSA total	1264
BSA HC pep	173
BSA LC pep	139
BSA HC HLA	629
BSA LC HLA	323
