# Supplementary material for: Structural engineering of chimeric antigen receptors targeting HLA-restricted neoantigens
Source: Nat Commun. 2021 Sep 6;12:5271. doi: 10.1038/s41467-021-25605-4 (PMC8421441; doi:10.1038/s41467-021-25605-4)
Supplement: Supplementary file 3 — Reporting Summary [file 41467_2021_25605_MOESM3_ESM.pdf]

nature research

Corresponding author(s): Bert Vogelstein, Shibin Zhou, Sandra B. Gabelli

Last updated by author(s): Jun 13, 2021

Reporting Summary

Nature Research wishes to improve the reproducibility of the work that we publish. This form provides structure for consistency and transparency in reporting. For further information on Nature Research policies, see our Editorial Policies and the Editorial Policy Checklist.

Statistics

For all statistical analyses, confirm that the following items are present in the figure legend, table legend, main text, or Methods section.

n/a	Confirmed	
☐	☒	The exact sample size (n) for each experimental group/condition, given as a discrete number and unit of measurement
☐	☒	A statement on whether measurements were taken from distinct samples or whether the same sample was measured repeatedly
☒	☐	The statistical test(s) used AND whether they are one- or two-sided<br>*Only common tests should be described solely by name; describe more complex techniques in the Methods section.*
☒	☐	A description of all covariates tested
☒	☐	A description of any assumptions or corrections, such as tests of normality and adjustment for multiple comparisons
☐	☒	A full description of the statistical parameters including central tendency (e.g. means) or other basic estimates (e.g. regression coefficient) AND variation (e.g. standard deviation) or associated estimates of uncertainty (e.g. confidence intervals)
☒	☐	For null hypothesis testing, the test statistic (e.g. F, t, r) with confidence intervals, effect sizes, degrees of freedom and P value noted<br>*Give P values as exact values whenever suitable.*
☒	☐	For Bayesian analysis, information on the choice of priors and Markov chain Monte Carlo settings
☒	☐	For hierarchical and complex designs, identification of the appropriate level for tests and full reporting of outcomes
☒	☐	Estimates of effect sizes (e.g. Cohen's d, Pearson's r), indicating how they were calculated

Our web collection on statistics for biologists contains articles on many of the points above.

Software and code

Policy information about availability of computer code

Data collection	LSDCGui (Life Science Data Collection software) version 1.0<br>iQue Forecyt 7.0<br>BD FACSDiva 6.0
Data analysis	coot 0.8<br>XDS Version January 26, 2018<br>PyMOL 2.4.2<br>CCP4 suite v7.0 (includes MolRep, PHASER, Refmac5, CONTACT)<br>SBGrid (includes Biologic Suite)<br>Biacore Insight Evaluation Software<br>Seq2Logo 2.0<br>iQue Forecyt 7.0<br>BD FACSDiva 6.0<br>FlowJo v9

For manuscripts utilizing custom algorithms or software that are central to the research but not yet described in published literature, software must be made available to editors and reviewers. We strongly encourage code deposition in a community repository (e.g. GitHub). See the Nature Research guidelines for submitting code & software for further information.

Data

Policy information about availability of data

All manuscripts must include a data availability statement. This statement should provide the following information, where applicable:

- Accession codes, unique identifiers, or web links for publicly available datasets
- A list of figures that have associated raw data
- A description of any restrictions on data availability

The final coordinates of IDH2R140Q-HLA-B*07:02, IDH2WT-HLA-B*07:02, 2Q1-Fab/IDH2R140Q-HLA-B*07:02, and 2Q1-Fab have been deposited in the PDB with accession codes 6UJ7 (http://doi.org/10.2210/pdb6UJ7/pdb), 6UJ8 (http://doi.org/10.2210/pdb6UJ8/pdb), 6UJ9 (http://doi.org/10.2210/pdb6UJ9/pdb) and 7KGU (http://doi.org/10.2210/pdb7KGU/pdb), respectively.

Field-specific reporting

Please select the one below that is the best fit for your research. If you are not sure, read the appropriate sections before making your selection.

☒ Life sciences ☐ Behavioural & social sciences ☐ Ecological, evolutionary & environmental sciences

For a reference copy of the document with all sections, see nature.com/documents/nr-reporting-summary-flat.pdf

Life sciences study design

All studies must disclose on these points even when the disclosure is negative.

Sample size	No sample-size calculations were performed and no statistical methods were employed to determine sample size. Non-structural data was obtained with either three or more biological or technical replicates which we deemed to be appropriate based on previous published studies in the field for establishing reproducibility and proof-of-concept.
Data exclusions	No data were excluded from the analyses.
Replication	The replication number is indicated in the figure legend of the corresponding figures where applicable. All attempts at replication were successful.
Randomization	Randomization was not relevant to this particular study. For the purpose of in vitro assays, all efforts were taken to minimize variability associated with the spatial location of a given well; thus, we felt that randomization was not necessary in this particular context. All experiments were performed in vitro with immunological and structural/molecular/cell biology techniques whereby the investigator designs, performs, and analyzes said experiment.
Blinding	Blinding was not relevant to this study. All experiments were performed in vitro with immunological and structural/molecular/cell biology techniques whereby the investigator designs, performs, and analyzes said experiment. As the same investigator designed, performed, and analyzed the experiments described herein, blinding was not possible.

Reporting for specific materials, systems and methods

We require information from authors about some types of materials, experimental systems and methods used in many studies. Here, indicate whether each material, system or method listed is relevant to your study. If you are not sure if a list item applies to your research, read the appropriate section before selecting a response.

Materials & experimental systems

n/a	Involved in the study	
☐	☒	Antibodies
☐	☒	Eukaryotic cell lines
☒	☐	Palaeontology and archaeology
☒	☐	Animals and other organisms
☒	☐	Human research participants
☒	☐	Clinical data
☒	☐	Dual use research of concern

Methods

n/a	Involved in the study	
☒	☐	ChIP-seq
☐	☒	Flow cytometry
☒	☐	MRI-based neuroimaging

Antibodies

Antibodies used	- Purified anti-human HLA-A,B,C Antibody [clone W6/32]: BioLegend #311402<br>- fd/M13 bacteriophage Antibody [polyclonal]: Novus Biologicals #NB100-1633<br>- PE Donkey anti-rabbit IgG (minimal x-reactivity) Antibody [polyclonal Poly4064]: BioLegend #406421<br>- HRP Anti-DDDDK tag (Binds to FLAG® tag sequence) antibody [clone M2]: Abcam #ab49763

- Goat anti-Human IgG (Gamma chain) Cross-Adsorbed Secondary Antibody, HRP [polyclonal]: ThermoFisher Scientific #62-8420
- Goat anti-Rabbit IgG (H+L) Secondary Antibody, HRP: ThermoFisher Scientific #65-6120
- APC anti-human IgG Fc Antibody [clone HP6017]: BioLegend #409306

Validation

All antibodies used are well-validated and highly-cited. Please see below for links to the manufacturer's page for details with regards to their validation methodology and data, as well as relevant citations:
- Purified anti-human HLA-A,B,C Antibody [clone W6/32]:
  --> https://www.biolegend.com/en-us/products/purified-anti-human-hla-a-b-c-antibody-1874
- fd/M13 bacteriophage Antibody [polyclonal]:
  --> https://www.novusbio.com/products/fd-m13-bacteriophage-antibody_nb100-1633
- PE Donkey anti-rabbit IgG (minimal x-reactivity) Antibody [polyclonal Poly4064]:
  --> https://www.biolegend.com/en-ie/products/pe-donkey-anti-rabbit-igg-minimal-x-reactivity-9751
- HRP Anti-DDDDK tag (Binds to FLAG® tag sequence) antibody [clone M2]:
  --> https://www.abcam.com/hrp-ddddk-tag-binds-to-flag-tag-sequence-antibody-m2-ab49763.html
- Goat anti-Human IgG (Gamma chain) Cross-Adsorbed Secondary Antibody, HRP [polyclonal]:
  --> https://www.thermofisher.com/antibody/product/Goat-anti-Human-IgG-Gamma-chain-Cross-Adsorbed-Secondary-Antibody-Polyclonal/62-8420
- Goat anti-Rabbit IgG (H+L) Secondary Antibody, HRP:
  --> https://www.thermofisher.com/antibody/product/Goat-anti-Rabbit-IgG-H-L-Secondary-Antibody-Polyclonal/65-6120
- APC anti-human IgG Fc Antibody [clone HP6017]:
  --> https://www.biolegend.com/en-us/products/apc-anti-human-igg-fc-7789

Eukaryotic cell lines

Policy information about cell lines

Cell line source(s)

RPMI-6666 (CCL-113), COS-7 (CRL-1651), and T2 (CRL-1992) cells were obtained from American Type Culture Collection (ATCC). T2A3 cells, an engineered cell line derivative of T2 cells (CRL-1992, ATCC), were a kind gift from Eric Lutz and Elizabeth Jaffee (JHU).

Authentication

Authentication was not performed on COS-7 and T2 cells as experiments were conducted shortly after thawing the stock vial directly acquired from ATCC. Engineered RPMI-6666 cells were authenticated by short-tandem repeat (STR) profiling, and parental origin (100% exact match of 8 core STR loci) was confirmed by ATCC Cell Line Authentication Service. T2A3 cells, provided by collaborators, have been extensively characterized in previously published reports (Anderson et al., Journal of Immunology, 1993; Thomas et al., Journal of Experimental Medicine, 2004) and therefore were not authenticated.

Mycoplasma contamination

All cell lines tested negative for mycoplasma contamination.

Commonly misidentified lines
(See ICLAC register)

No commonly misidentified lines were employed in this study.

Flow Cytometry

Plots

Confirm that:

☒ The axis labels state the marker and fluorochrome used (e.g. CD4-FITC).

☒ The axis scales are clearly visible. Include numbers along axes only for bottom left plot of group (a 'group' is an analysis of identical markers).

☒ All plots are contour plots with outliers or pseudocolor plots.

☒ A numerical value for number of cells or percentage (with statistics) is provided.

Methodology

Sample preparation

Flow cytometry analysis was performed only on cultured cancer cell lines.

Instrument

- LSRII cytometer (BD Biosciences)
- IntelliCyt iQue Screener PLUS (Sartorius)

Software

FACSDiva & FlowJo software (BD Biosciences); iQue Forecyt (Sartorius)

Cell population abundance

No sorting was performed.

Gating strategy

Cells were gated for live/viable singlet cells based upon standard practice FSC/SSC gating strategies.

☒ Tick this box to confirm that a figure exemplifying the gating strategy is provided in the Supplementary Information.
